# Changes in Body Mass Index across Age Groups in Iranian Women: Results from the National Health Survey

**DOI:** 10.1155/2012/848403

**Published:** 2012-02-08

**Authors:** Enayatollah Bakhshi, Behjat Seifi, Akbar Biglarian, Kazem Mohammad

**Affiliations:** ^1^Department of Statistics and Computer, University of Social Welfare and Rehabilitation Sciences, Tehran, Iran; ^2^Department of Physiology, Medicine School, Tehran University of Medical Sciences, Tehran, Iran; ^3^Department of Biostatistics, School of Public Health and Institute of Public Health Research, Tehran University of Medical Sciences, Tehran, Iran

## Abstract

*Background*. To investigate the associations between some factors with weight gain across age groups in Iranian women. *Methods*. Proportional odds model was used to estimate the probability of BMI categorized as a function of education, economic index, workforce, smoking, marital status, and place of residence adjusted for age, using data from the “National Health Survey in Iran” database. It included 14176 women aged 20–69 years. *Results*. For all covariates, age was directly associated with overweight and obesity before 60 years of age. Among women aged 20–40 years, the rates of change in probabilities of overweight and obesity were highest. Among women, being inactive, with high economic index, married, being nonsmoker, in an urban residence, with lower educational attainment, all increased the probabilities of overweight and obesity. *Conclusions*. Women aged 20–40 years gained weight faster than other groups. They may need additional information and more support on how to reduce their risk for weight gain through positive health behaviors.

## 1. Introduction

Overweight and obesity are as public health problems, due to both their rapid growth in recent decades and to their related health disorders, such as cardiovascular diseases, diabetes, some cancers, and other diseases [1–8]. Studies have also showed relationships between obesity and chronic pain [9] and Alzheimer's disease [10]. 

In 2002, nearly half a billion of the world population considered to be overweight or obese [11]. Obesity is assuming epidemic proportions in both developed and developing countries [12–15]. In 2003-04, 17.1% of US children and adolescents were overweight and 32.2% of adults were obese [16]. Almost one third of adult Canadians are at increased risk of disability, disease, and premature death due to obesity [17]. Obesity is relatively common in Europe, especially in southern and eastern countries, and studies from repeated surveys suggest that the prevalence of obesity has been increasing last years [18]. Up to 2000, the prevalence of obesity in Western countries was suggested to vary between 15 percent and 20 percent [19].

In recent years, the statistics were appalling. The prevalence of obesity in USA, Canada, Australia, United Kingdom, Iran, and Egypt was 31.8, 24.3, 25.1, 24.9, 21.6, and 34.6, respectively [20]. Overall, more than one out of ten of the world adult population was obese. Nearly 1.5 billion adults, 20 and older, are now considered to be overweight or obese. Of these, nearly 300 million women and more than 200 million men were obese [21]. In 2010, almost 43 million children (35 million in developing countries and 8 million in developed countries) were estimated to be overweight or obese [22].

Most studies have investigated the relationship between sociodemographic factors and obesity. It has found a significant association between weight gain and aging [23–26]. 

Sobal and Stunkard [27] found a strong inverse relationship between socioeconomic status and obesity in women in affluent societies, with a higher proportion of obese women in lower socioeconomic groups. In low-income countries, obesity is more common among middle-age women, people of higher socioeconomic status, and people living in urban communities [28, 29].

Although the association of overweight with smoking, alcohol consumption, dietary habits, and physical activity has been analyzed in many studies, the findings are not consistent. Wilsgaard et al. [30] showed that being a smoker is associated with lower BMI values. 

We aimed to assess the associations between some factors with weight gain across age groups among women by using cross-sectional data from the National Health Survey in Iran (NHSI).

## 2. Material and Methods

### 2.1. Study Population

The National Health Survey in Iran (NHSI) is a survey designed to gain comprehensive knowledge and information about health care problems and difficulties throughout the country, 1999-2000. The survey was conducted under the supervision and with the financial support of the Iranian Ministry of Health and Medical Education. The population sample of the survey consisted of one thousandth of the total Iranian population; non-Iranian were excluded. They were randomly chosen by cluster sampling. Each cluster comprises of 8 households. The choice of 8 households for the cluster size was based on one-day performance capacity of the data collection group: four persons (2 physicians, 1 interviewer, and 1 lab technician). The statistical framework was based on the household lists available with every Health Department in the provinces, usually updated annually. Data from the National Health Survey were considered in this investigation. In this study, 14176 women, 8957 urban, and 5219 rural aged 20–69 years were investigated. These data were collected by the National Research Center of Medical Sciences and are presented partially at the Department of Biostatistics and Epidemiology/Tehran University of Medical Sciences for research [31]. We excluded pregnant women from the analyses. This study was approved by the Ethic Committee of the University of Social Welfare and Rehabilitation Sciences.

### 2.2. Measurements

#### 2.2.1. Response Variable

Height and weight were measured rather than self-reported. Height was measured without shoes to the nearest 5 mm. Weight was measured to the nearest 0.1 kg with the subject in light indoor clothes, with emptied pockets and without shoes. BMI (body mass index) was calculated as weight in kilograms divided by square of height in meters, squared, and subjects were classified into underweight defined as BMI < 18.5, normal weight as BMI 18.5–24.9, overweight as BMI 25.0–29.9, and obese as BMI ≥ 30.

#### 2.2.2. Independent Variables

**Age**

Information about the respondent age was based on their self-reported birth year, and subjects were stratified into five 10-year age groups (20–29, 30–39, 40–49, 50–59, and 60–69 years).

**Education**

Educational level was measured in years of school. Years of schooling were divided into three groups: person with basic (0–8 years), moderate (9–12 years), or high (more than 12 years) education.

**Economic Index**

Due to ethical considerations, we did not ask respondents about their income. Because they were afraid of paying their taxes. We surrogated economic index for their household income. Economic index was defined as square meter of living place divided by number of household. Participants were classified by their economy index status into four classes: (1) low (economic index ≤ Quartile 1), (2) lower-middle (Quartile 1 < economic index ≤ Quartile 2), (3) upper-middle (Quartile 2 < economic index ≤ Quartile 3), and (4) high (economic index > Quartile 3).

**Place of Residence**

The subjects were grouped according to their place of residence as living in cities (urban) or villages (rural).

**Workforce**

Active workforce was defined as the part of the female population that belongs to the currently employed (as employees) or self-employed category as opposed to inactive workforce (being a housewife/houseworker, pensioner, student, or unemployed).

**Smoking**

Smoking status was dichotomized into smoker (those who smoke every day and have smoked at least 100 cigarettes in their lives) versus nonsmoker (others).

**Marital Status**

To make the marital status variable, it was dichotomized into legally married and nonmarried groups.

Note that we have no information on household income and physical activity, but economic index is surrogate for household income and we used workforce factor. Due to ethical considerations, we did not ask respondents about their income, because they were afraid of paying their taxes. The consumption of alcohol is prohibited in Iran. Therefore, there were no information on alcohol consumption.

### 2.3. Statistical Analysis

We used proportional odds model to assess the influence of the independent variables listed previously on the probability of obesity and overweight. We carried out score tests for the proportional odds assumption, which was found to hold. In addition, we tested the interaction terms using reduced models excluding nonsignificant terms. For obesity and overweight, odds ratios and 95% confidence intervals were calculated. For all covariates, we calculated the probability of obesity and overweight across age groups. All analyses were performed using SAS software, version 9.1 for windows.

## 3. Results

The mean BMI of women was 25.33 kg m^−2^ (95% CI: 25.25–25.41). Prevalence (%) of the body mass index levels according to independent variables was assessed (Table 1). Table 1 shows that obesity is much more prevalent among women aged 40–60 years, less educated, high economic index, inactive workforce, nonsmoker, married, and resident in city.

We started by fitting a preliminary proportional odds model including only age to observe the influence of the potential confounders on overweight and obesity. Unadjusted odds ratios were 2.50, 3.41, 3.52, and 2.45 for age groups 30–40, 40–50, 50–60, and 60–69 years, respectively.

**Figure 1:**
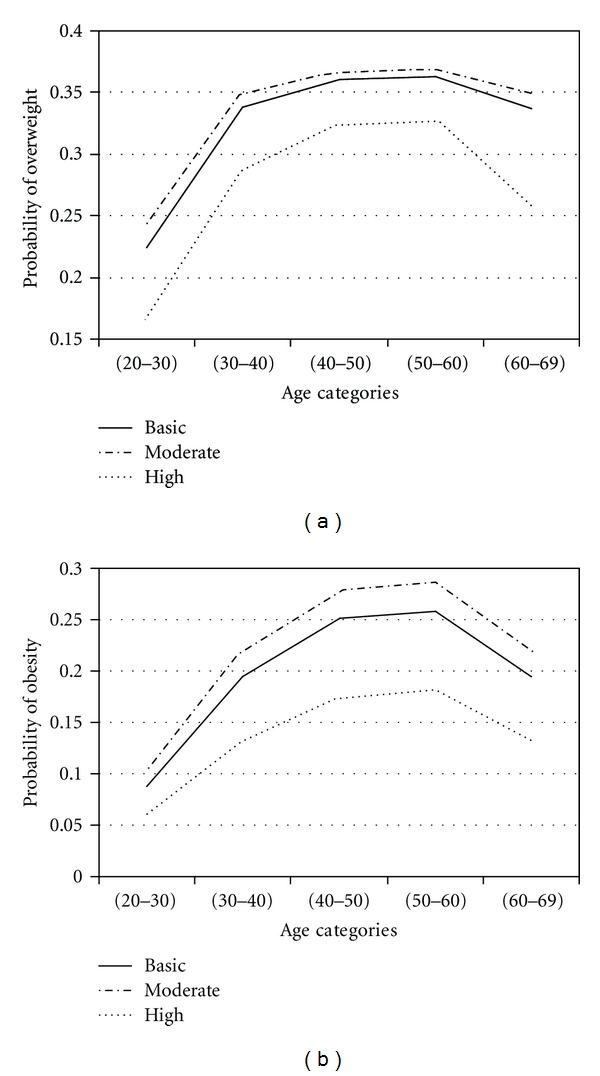
Probabilities of overweight and obesity across age groups for education level.

**Figure 2 fig2:**
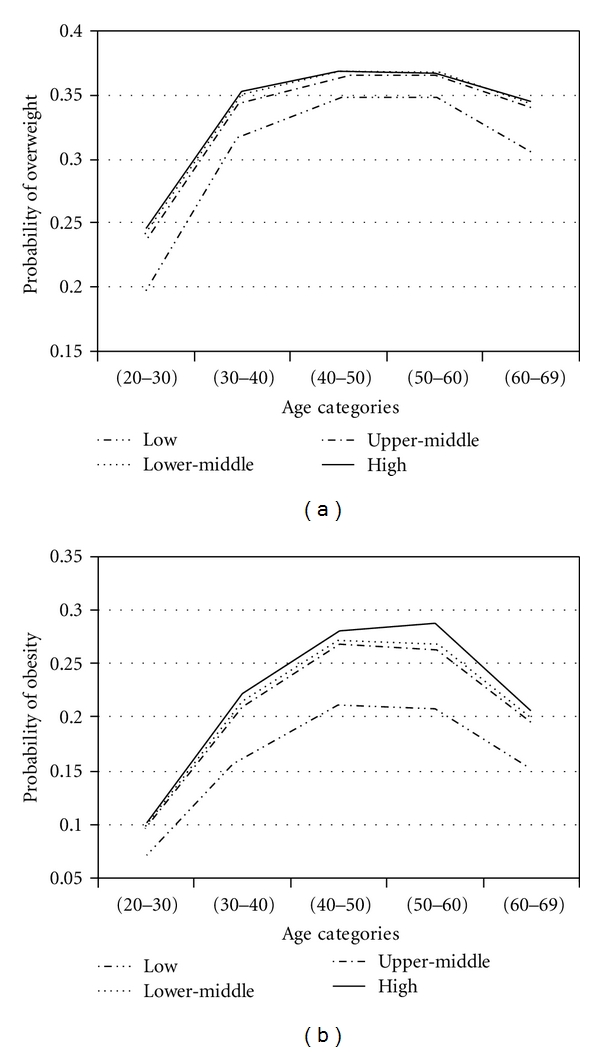
Probabilities of overweight and obesity across age groups for economic index.

**Figure 3 fig3:**
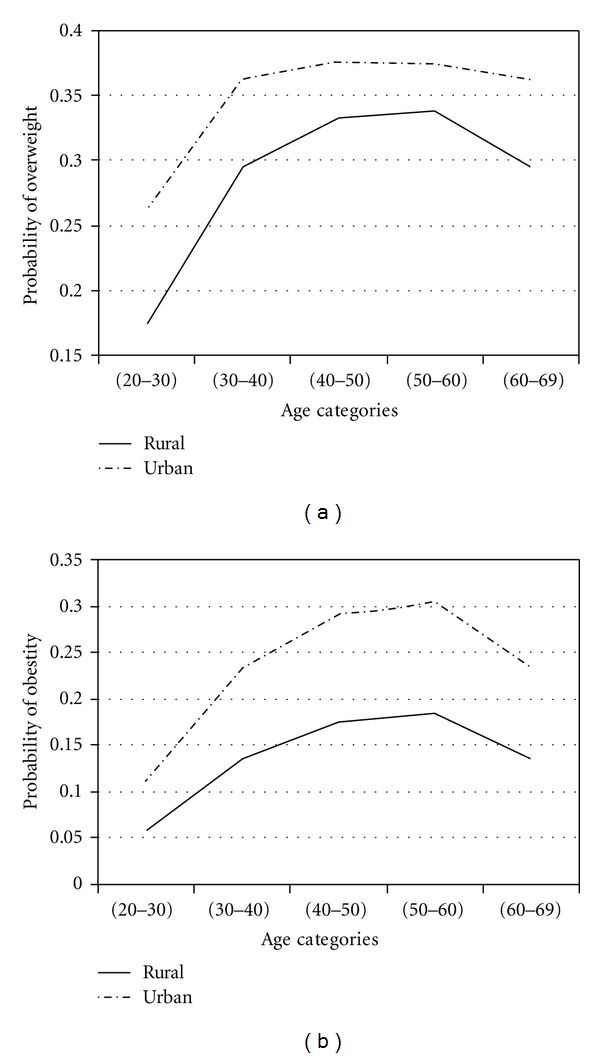
Probabilities of overweight and obesity across age groups for place of residence.

**Figure 4 fig4:**
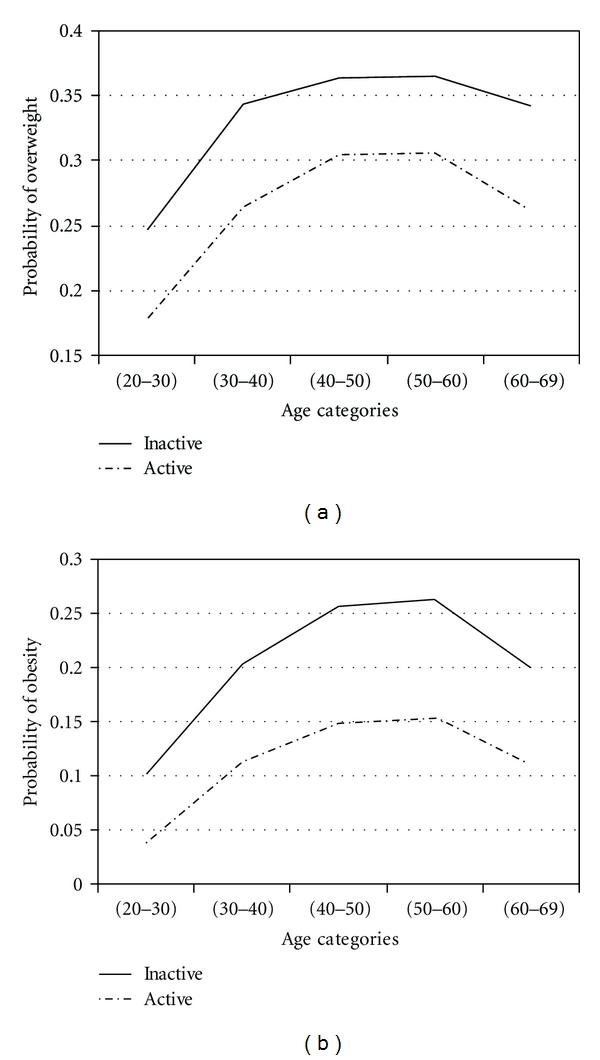
Probabilities of overweight and obesity across age groups for workforce level.

**Figure 5 fig5:**
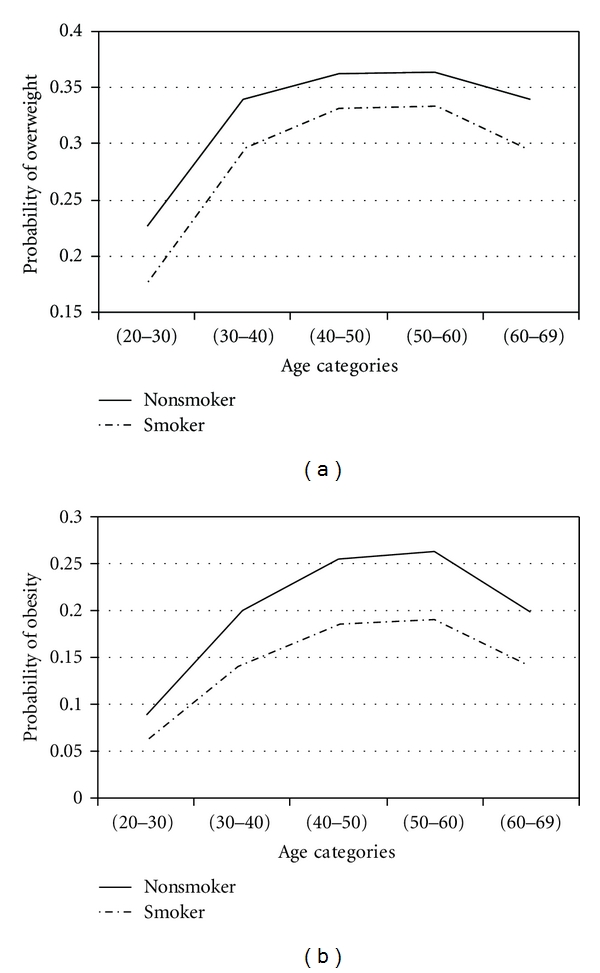
Probabilities of overweight and obesity across age groups for smoking status.

**Figure 6:**
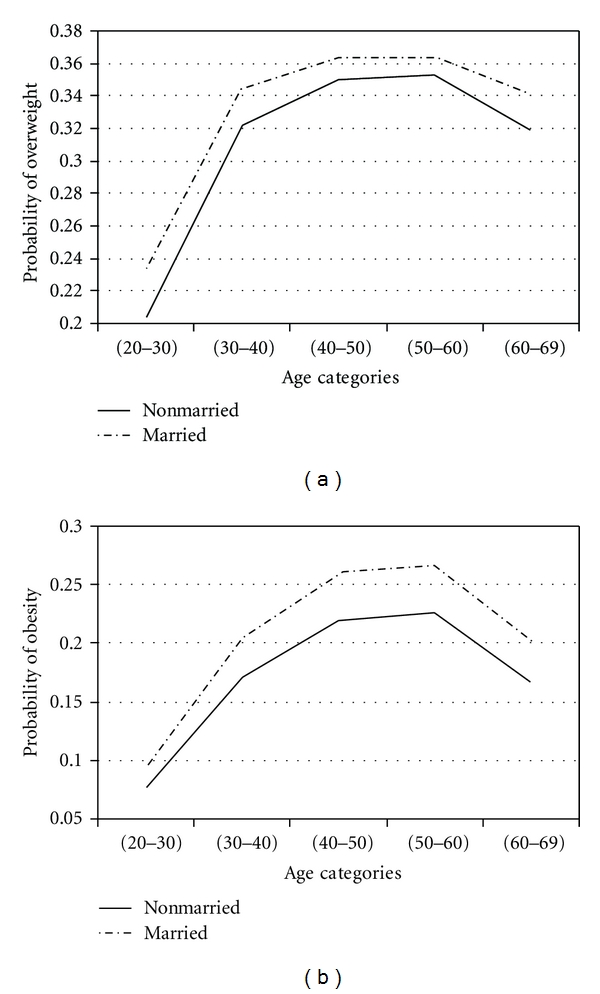
Probabilities of overweight and obesity across age groups for marital status.

To assess effects of factors in obesity and overweight across age groups, adjustment for age was performed by using proportional odds model. With four response categories, the model had three intercepts. They are of interest for computing response probabilities. For all covariates, the probability of overweight and obesity was calculated. The results are presented in Figures 1 [Fig fig2] [Fig fig3] [Fig fig4] [Fig fig5]–6.

Figures 1 [Fig fig2] [Fig fig3] [Fig fig4] [Fig fig5]–6 show that age was directly associated with overweight and obesity before 60 years of age. In other words, the probabilities of overweight and obesity increase from 20 to about 50 years of age and decrease after age 60 years. The curves are either flat or in increase in the 50–60 age group. We observe rise in a very high rate among women aged 20–40 years.

We used proportional odds model including age, economic index, workforce status, education level, place of residence, smoking status, and marital status. All were significantly associated with underweight, normal weight, overweight, and obesity. Odds ratios and 95% confidence intervals were calculated (Table 2). 

Table 2 shows that among women, being inactive, with high economic index, married, being nonsmoker, in an urban residence, with lower educational attainment, all increased the probability of overweight and obesity. Younger age decreased the probability of overweight and obesity. The odds ratios were 2.16 (95% CI: 1.96–2.35), 2.93 (95% CI: 2.67–3.23), 2.96 (95% CI: 2.63–3.32), and 1.99 (95% CI: 1.76–2.25) for age groups 30–40, 40–50, 50–60, and 60–69 years, respectively.

 For overweight and obese participants with moderate and high education, odds ratios were 0.90 (95% CI: 0.83–0.98) and 0.57 (95% CI: 0.47–0.69), respectively. 

Women with high economic index were more likely to be overweight and obese. The odds ratios for overweight and obese participants with lower-middle, upper-middle, and high levels were 1.37 (95% CI: 1.26–1.50), 1.38 (95% CI: 1.27–1.51), and 1.49 (95% CI: 1.36–1.63), respectively.

We observed an association between workforce level and BMI groups. For active participants odds ratio was 0.57 (95% CI: 051–0.64).

Overall, nonsmokers were more obese. For smokers, odds ratio was 0.68 (95% CI: 0.54–0.85).

Among women, the odds for urban was 1.97 times that for rural (95% CI: 1.84–2.11). 

Married women were more obese. Odds ratio was 1.19 (95% CI: 1.10–1.29).

## 4. Discussion

Concern about the increased prevalence of overweight and obesity has heightened interest in the association between some factors with weight gain across age groups. In this cross-sectional study we found probabilities of overweight and obesity by identifying a variety of factors that are associated with weight gain. Overall, probability of overweight is higher than probability of obesity. Age was directly associated with overweight and obesity before 60 years of age. The probabilities of overweight and obesity initially increased, and these probabilities decreased for women aged more than 60 years. It is possible that weight loss among older women is result of medical advice to control or prevent obesity-related chronic diseases. For all covariates, the highest probabilities of overweight and obesity were among women aged 50–60 years. Among women aged 20–40 years, the rates of change in probabilities of overweight and obesity were highest. The mechanism of weight gain in women aged 20–40 years is likely multifactorial; that is, younger people love fast food and their spouses have to follow them. An increased consumption of fast foods by young adults has been repeatedly shown to be associated with obesity and excess weight gain [32]. Although some studies showed that any association between number of children and weight gain is a result of lifestyle and behaviors associated with family life rather than being as result of the biological impact of pregnancy in women [31, 33], it may include physiological mechanisms in the women, especially after their pregnancy. Prevention of weight gain among women aged 20–40 years may have a significant public health impact [34] and further work is needed to understand these relationships.

**Education**

Women with moderate education had higher probabilities of overweight and obesity than high educated women. Our results are consistent with some studies [26, 35–38]. Note that the women with moderate education had higher probabilities of overweight and obesity than basic educated women. The differences in probabilities of overweight and obesity between high educated and two other levels were noticeable. Higher education may provide knowledge or resource influences on weight loss.

**Economic Index**

Many studies have found an inverse relation between socioeconomic level and weight [39–45]. It is not straightforward matter to compare those results with ours, because of the different study designs, time span, different region, and method of analysis. In our study, subjects with high level had higher probabilities of overweight and obesity than the other level. These results are consistent with the findings of some study in developing country [46]. In Iran, economy, business, social affair, and so forth are controlled by some people named Bazarry. These people have usually low education. Higher economy may not provide knowledge or resource influences on weight loss.

**Residence**

Urban women had higher probabilities of overweight and obesity than rural women. Among urban women, the probability of overweight initially increased and then changes were fairly slow but this decreasing was sharply in rural women aged >60 years. Among women aged 50–60 years, the probability of obesity for urban was approximately 2 times that for rural.

**Workforce**

Inactive women had higher probabilities of overweight and obesity than active women. Our results are consistent with some studies. For example, Swedish women who returned to work soon after pregnancy also retained less weight than women who stayed at home [47, 48]. Obesity may be more acceptable among unemployed persons. It is also possible that there is more discrimination against the obese, or obese women may end up in lower status jobs through stronger selective processes in Iran. Another explanation for the effect of the workforce may involve energy expenditure at work or the structured lifestyle that active woman imposes.

**Smoking**

The increase in body weight by age was found to be lower among smokers than among nonsmokers. Biological mechanisms as well as psychological factors may be involved. An increase of energy expenditure while smoking, both in resting and in light physical activity conditions, may relate to weight loss in smokers. Our results are consistent with the findings of most studies [49–53].

**Marital Status**

Most studies showed that marriage is associated with weight gain [54, 55]. The finding in this study showed that women who were married tended to gain more weight across the age than those who were not married. The rate of change in probability of overweight in aged <40 years was noticeable. It is possible that the presence of a spouse may operate as a social factor on weight gain.

In Iran, it is commonly believed that overweight and obese people are lazy and gluttonous and they lack self-control. Many obese people do not go out in public because the devices are too uncomfortable. For example, they cannot go to the movies because the seats are too small. Obese people are also more likely to lose the benefits of exercise and it may cause further weight gain. They often feel inferior to others because many people would not to be friends with an obese person. They often get disapproving stares from others. Some people believe that an obese person is taking up more space than he or she should and a job is often denied because of their weights.

**Limitations and Strength**

Some limitations of this study should be noted. Cause-and-effect cannot be inferred from our cross-sectional data. However, this should be confirmed by further longitudinal studies. It is a limitation that in this study marital status could be categorized into legally married and nonmarried only. Nonmarried people are also a very heterogeneous group and should be more closely examined in further studies. Unfortunately, income and physical activity were not used in our investigation.

Strengths of this study include the national random sample with a considerable age range and a BMI measurement that has been shown to be more valid than self-report measures on body weight and height. Obese people tend to underreport their BMI whereas thin people do the reverse [56, 57].

## 5. Conclusions

Increases in response were observed through the 20–60 years; however, all age beyond 60 years result in a decrease in probabilities of overweight and obesity. Women aged 20–40 years gained weight faster than other groups. They may need additional information and more support on how to reduce their risk for weight gain through positive health behaviors.

## Figures and Tables

**Table 1:** Prevalence (%) of underweight, normal weight, overweight, and obesity according to sociodemographic and smoking in a random sample of 14176 women in Iran, 1999-2000.

Variable	Underweight	Normal weight	overweight	obese
Age group (years)				
20–30	10.5	57.8	22.6	9.1
30–40	4.0	42.4	33.5	20.1
40–50	3.2	35.8	35.2	25.8
50–60	2.6	35.0	37.1	25.3
60–69	4.4	41.4	36.2	18.0

Education level				
Basic	5.8	44.6	30.8	18.8
Moderate	6.5	47.9	30.9	14.6
High	11.0	63.4	19.9	5.7

Economic index				
Low	8.0	50.6	27.2	14.2
Lower-middle	5.5	44.2	30.9	19.4
Upper-middle	5.4	45.0	31.5	18.1
High	5.2	42.9	32.9	19.0

Workforce				
Inactive	5.2	44.0	31.8	18.9
Active	13.1	61.0	20.0	6.0

Smoking status				
Nonsmoker	6.1	45.9	30.5	17.5
Smoker	6.6	46.5	31.1	15.8

Marital status				
Non-married	8.1	49.4	29.9	12.6
Married	5.6	45.0	30.6	18.8

Place of residence				
Rural	8.3	54.4	25.8	11.5
Urban	4.8	41.0	33.2	21.0

**Table 2:** Adjusted* odds ratios for the likelihood of being overweight and obese^§‡^, by sociodemographic and smoking among random sample of 14176 Iranian women in the proportional odds model, 1999-2000.

Variable	OR^#^	95% CI^†^
Age group (years)		
20–30	1.00	
30–40	2.16	1.96–2.35
40–50	2.93	2.67–3.23
50–60	2.96	2.63–3.32
60–69	1.99	1.76–2.25

Education level		
Basic	1.00	
Moderate	0.90	0.83–0.98
High	0.57	0.47–0.69

Economy index		
Low	1.00	
Lower-middle	1.37	1.26–1.50
Upper-middle	1.38	1.27–1.51
High	1.49	1.36–1.63

Place of residence		
rural	1.00	
urban	1.97	1.84–2.11

Workforce		
Inactive	1.00	
Active	0.57	0.51–0.64

Smoking status		
Nonsmoker	1.00	
Smoker	0.68	0.54–0.85

Marital status		
Non-married	1.00	
Married	1.19	1.10–1.29

*Adjusted for all other variables.

^§^Obese, BMI ≥ 30; overweight BMI 25.0–29.9.

^‡^BMI: body mass index (weight (kg)/height (m)^2^).

^#^OR: odds ratio.

^†^CI: confidence interval.
